# In search of factors related to migration affecting children’s health – an analysis of documents guiding health visits within the Swedish school health services

**DOI:** 10.1186/s13690-023-01125-z

**Published:** 2023-06-13

**Authors:** Emmie Wahlström, Marie Golsäter, Inger K. Holmström, Peter Larm, Maria Harder

**Affiliations:** 1 ChiP Research Group, School of Health, Care and Social Welfare, Mälardalen University, Västerås, Sweden; 2 School of Health, Care and Social Welfare, Mälardalen University, Box 883, 721 23 Västerås, Sweden; 3 CHILD-Research Group, School of Health and Welfare, Jönköping University, Jönköping, Sweden; 4 Child Health Services and Futurum, Region Jönköping County, Jönköping, Sweden; 5 Department of Public Health and Caring Sciences, Uppsala University, Uppsala, Sweden; 6 Department of Public Health Sciences, Stockholm University, Stockholm, Sweden; 7 Child Health Care Services, Region Västmanland, Västerås, Sweden

**Keywords:** Guidelines, Children, Migration, Health disparities, Health determinants, School nursing, Document analysis, Evidence-based practice

## Abstract

**Background:**

Migration affects the health of children worldwide. Therefore, school nurses who encounter these children as part of their everyday practice need support from guidelines on how to promote the health of children who have migrated or whose parents have migrated. Yet knowledge regarding such content in guidelines of school nursing practice is sparse. Therefore, this study aims to investigate how municipal and regional guidelines and health questionnaires used in health visits in the Swedish school health services include factors related to migration that affect children’s health.

**Methods:**

A document analysis of municipal and regional guidelines and health questionnaires guiding school nurses’ practice in health visits was conducted during the autumn of 2020. In total, 687 guidelines and health questionnaires were analyzed using deductive content analysis.

**Results:**

The results show that municipal and regional guidelines and health questionnaires used in health visits in the Swedish school health services include content on many factors related to migration that affect children’s health. Yet the content was limited, and none was found on factors related to discrimination based on ethnicity or origin.

**Conclusion:**

Guidance related to promoting the health of children who have migrated or whose parents have migrated should include all factors affecting these children’s health. Therefore, to strengthen school nurses’ evidence-based practice, guideline development might be needed, although guidelines and health questionnaires exist and include content on many factors related to migration affecting the health of children in order to provide equitable healthcare for all children, regardless of country of origin.

**Supplementary Information:**

The online version contains supplementary material available at 10.1186/s13690-023-01125-z.

**Table:** 

**Text box 1. Contributions to the literature**	
• Research has shown that guidelines are important for school nurses’ clinical practice. Yet little research has focused on the content in these guidelines, especially for encounters where children having migrated meet school nurses. • The results show that documents guiding school nurses work provides some directions on factors affecting children’s health related to migration, but also that factors related to discrimination based on ethnicity or origin are not included. • These findings contribute to highlight shortages in school nurses’ guidelines when encountering children having migrated as well as provides grounds for continued improvement of clinical guidelines.	

## Background

Migration is a global phenomenon that affects the health and living conditions of children worldwide. For children who have migrated or whose parents have migrated, previous research shows that a complex combination of factors affect children’s health and risks of ill-health, such as discrimination, poverty, education, comfort in school and among friends, exposure to infectious diseases, asylum processes, as well as parents’ well-being and health [1–8]. These factors exist pre, during and post migration and relate to the various conditions that children who have migrated and their families have lived in and live in. Migration is thus described as a process where the effects of migration on health can be imminent or occur some time after resettlement in a new country [1]. Given the major influence that migration has on health, migration has even been described as a key determinant for health inequality [9]. Thus, knowledge regarding factors influencing the health of children who have migrated or whose parents have migrated is of importance among health professionals such as school nurses, in order to promote these children’s health and provide equitable health care for all children.

Promotion of school-aged children’s health and prevention of ill-health, for example by screening for risks of ill-health, is a major assignment of school nurses working within the school health services (SHS) [10, 11]. This assignment is mainly carried out through regular one-on-one encounters i.e., health visits. In Sweden and other European countries, such health visits contain physical examinations of height, weight, vision and hearing as well as health promotion and prevention activities focusing on lifestyle, such as physical activity, nutrition and sleeping habits. Description of the content of health visits is included in written guidelines of school nurses' practice [11], which exist to ensure practice is evidence-based [12, 13]. Approximately 90 percent of all countries in Europe have legislation, policies or regulations that guide the provision of the SHS [14], thereby guiding school nurses’ practice in health visits. However, there have been calls to increase knowledge regarding migration and health, and a need of support from guidelines concerning the topic has been expressed [15–17].

Research has been conducted regarding the content of the SHS in varying countries [10, 18], the implementation of guidelines within various fields of school nursing practice [19–21] as well as evidence-based practice in relation to guidelines and guideline development in school nursing [22]. Yet research targeting whether guidelines include content related to migration in documents guiding school nursing practice is sparse. This is important, especially since migration trends have altered the composition of populations in various countries, increasing the number of children who have migrated [23, 24]. In Sweden, the proportion of children who were born abroad or who have two parents both born abroad is currently about 35 percent of all children [25]. A study among Swedish school nurses similarly shows that a majority of school nurses report encountering children who have migrated or whose parents have migrated at least on a weekly basis [26]. Thus, in order to promote health equality among children, it is important that written guidelines for school nursing practice in health visits also include factors related to migration that affect children’s health.

### Guidelines and health questionnaires for school nursing in Sweden

In Sweden, school nursing practice is guided by national SHS guidelines [11] as well as municipal or regional guidelines of SHS. The municipal and regional guidelines are based on the national guidelines but clarify or adapt the national guidelines to the local school nursing practice in the specific municipality or region [27, 28]. These municipal and regional guidelines often include both descriptions of how health visits should be conducted as well as what topics should be addressed in dialog with the child. The topics are based on the content of a health questionnaire distributed to the child before or at the health visit [29, 30]. The content of health questionnaires is also used as a guide to make sure that all important determinants of health are covered during the health dialog [29]. Studies of the content of health questionnaires have found that the content mainly relates to communication, mental health, the school environment, contacts with the health services, the prevalence of psychosomatic symptoms as well as lifestyle [31, 32]. Still, the content of the municipal and regional guidelines as well as the health questionnaires has been found to vary between municipalities [13, 30–33]. Recent studies [27, 28] also confirm this variation in content, yet these studies have not investigated the content or focused on migration and its various influences on health among children.

### Aim

This study aims to investigate how municipal and regional guidelines and health questionnaires used in health visits in the Swedish school health services include factors related to migration that affect children’s health.

## Methods

This study is a descriptive exploratory study of local documents (i.e., guidelines and health questionnaires formulated by a municipal SHS or regional collaboration of municipalities) guiding health visits in the Swedish school health services. Document analysis can be used as a standalone method to study the content of any type of document [34, 35].

### Setting

The Swedish SHS is school-based, free of charge, available for all school-aged children and mainly focuses on health-promoting and preventive actions [11]. School nurses are assigned to provide health promoting and preventive actions, by inviting children to health visits on at least four occasions during their school years. Children invited to health visits are 6, 10, 13–14 and 16 years old. In Sweden, health visits involve physical examinations of height, weight, vision, hearing and the spine as well as a health dialog, usually based on a health questionnaire regarding lifestyle, mental health, living conditions and the school situation [11, 30].

### Data collection

The data collection was conducted during October, 2020 to January, 2021. To identify documents, an e-mail describing the study was sent to the manager of the school health services in every municipality in Sweden (*n* = 290) asking for all local guidelines applicable to health visits and health questionnaires used by school nurses in the municipality. Two reminders were distributed with a two-week interval. In addition, the municipality website was searched for documents relevant to the study. In total, 176 municipalities responded to the e-mail, of which 142 municipalities replied (a response rate of 49%), describing the documents used and/or submitting materials. Response rates within each of Sweden’s 21 regions, consisting of one to several municipalities, varied between 25 and 100%. Responding municipalities were localized in the major city areas as well as the countryside from the north to the south of Sweden. In their replies, the municipalities sent everything from 181 individual documents to a short answer saying they followed the national guidelines or used the same guidelines or questionnaires as another municipality. In total, 1 899 documents were received from the municipalities. The mean value of received documents per municipality was 13 documents and the median was six documents.

### Document analysis

For this study, the methodological process of conducting the document analysis followed the READ approach created by Dalglish et al. [35]. The READ approach includes four phases: readying the material, extracting relevant data, analyzing data and distilling the findings. Using the READ approach provides a systematic procedure for document analysis, enhancing procedural rigor while accommodating a variety of research questions and documents from any level (local, regional, national, etc.).

#### Readying the material

In total, 2 037 documents were collected from websites and via e-mail from the municipalities. All documents were recorded in an Excel spreadsheet with information about: county, municipality, author, document title, audience/recipient, aim, date of production, context, source of document, type of document and whether it was to be included or excluded in further analysis. The exclusion process was conducted by skimming through the content of the documents using nine criteria of exclusion (see Table 1) based on the study aim.

**Table 1:** Criteria used in the exclusion of irrelevant documents

Criteria of exclusion	Clarification
Not possible to open document	The file or link is not possible to open or access
Document is not a local document	Excluded documents are authored by organizations or authorities at national level. Documents authored by regional organizations or in cooperation between several municipalities are included
Document is not relevant to the medical school health services	Is not explicitly relevant to the medical part of the school health services (i.e., school nursing, school physician, health examinations, etc.)
Document is not explicitly relevant to school nurses or children who have migrated or whose parents have migrated	Excluded documents focus on the work of other professionals (e.g. school physicians), other health care services, the facilities or equipment, or solely on parents or caregivers. Documents aimed at parents or caregivers but concerning the child are included
Document does not concern health visits, i.e., concern the other assignments or work tasks of the school nurse	Excluded documents concern: filing, archives, requisition of patient records, handling of referrals, conducting vaccination, handling of patient records, emergency care, handling of drugs and other pharmaceutical substances, secrecy, and self-care
Document not available in Swedish or English	The document is a translation of a similar written text in Swedish
Document does not provide descriptions of content in health visits (e.g. physical examinations, health dialogs, health topics discussed in health visits)	Excluded documents: documents where the terms ‘health visit’ or ‘health dialog’ are mentioned but there is no further description, documents concerning health examinations or issues not included in regular health visits. Included documents: descriptions of health visits, health dialogs, physical examinations and screening results, descriptions of what school nurses do during health visits, and materials about issues addressed in health dialogs (i.e., family, friends, lifestyle, female genital mutilation, sexuality, etc.)
Document is not a guideline	Excluded documents: invitations to health visits, feedback concerning results of health visits, information letters, brochures, conversation aides (bubbles, conversation maps, images, etc.) and regional or local reports on health issues
Document is not a health questionnaire used in health visits	Excluded documents: questionnaires that only contain consent forms and health questionnaires applicable when applying for certain types of education (vocational or athletic/sports)

**Fig. 1:**
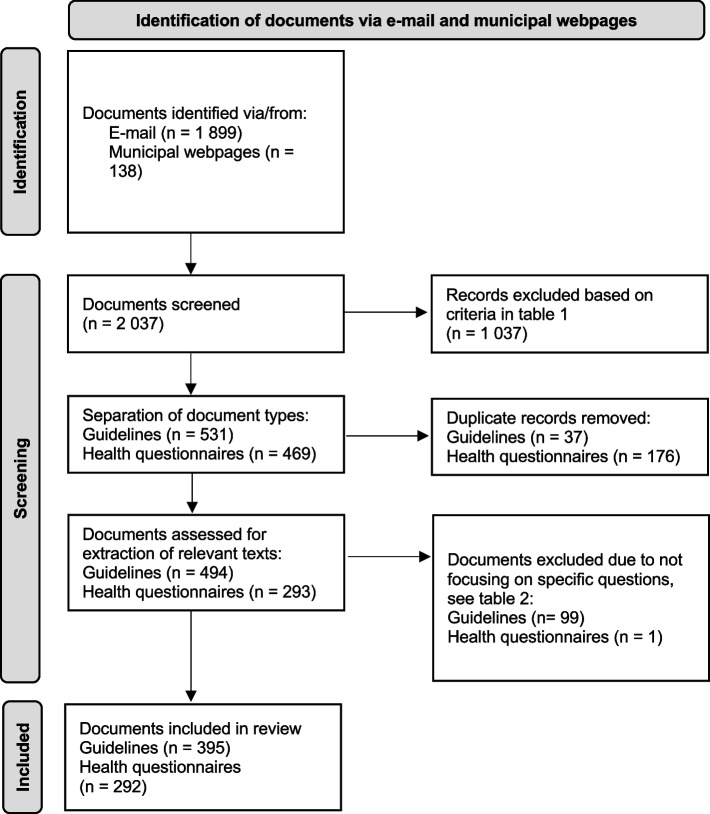
Flow chart of exclusion and inclusion of documents in the study

This process resulted in the exclusion of 1 037 documents (see Fig. 1). The remaining documents were organized according to the topic/theme of the document, such as physical examinations, health dialog and health problems. This organizing of documents facilitated identification of duplicates and included separating guidelines (*n* = 531) from health questionnaires (*n* = 469). As a result, the remaining documents included in this study consisted of 395 guidelines and 292 health questionnaires from 104 municipalities all over Sweden.

#### Extracting relevant data

As the document analysis focused on migration and its various influences on children’s health, factors influencing the health of children who had migrated or whose parents had migrated were sought and identified in previous reports and scientific articles [1–7, 36–45]. The list of identified factors in the cited research is available in Additional file 1. Based on the list of factors, a framework was created to facilitate the extraction process (see Additional file). The framework sorted the factors based on system level, from individual level to societal level, inspired by the Dahlgren and Whitehead model of main health determinants [46].

The extraction process was guided by four self-constructed questions (see Table 2). The 531 guidelines and 469 health questionnaires were read through by the first author (EW) and assessed in relation to the questions. Quotations of relevant texts were extracted and recorded in an Excel spreadsheet, along with answers to the extraction questions. This reading of documents resulted in the exclusion of 136 guidelines and 177 health questionnaires (see Fig. 1). To check for consistency in extraction, the last author (MH) independently read through 10 documents and extracted texts relevant to the questions. When compared with extractions by the first author in the same documents, the extractions made by both the first (EW) and the last author (MH) were identical. In addition, the first (EW) and last authors (MH) discussed the exaction process continuously.

**Table 2:** Questions used to guide the extraction of relevant text extracts from the documents

Does the document include a factor in the framework of factors related to migration affecting children’s health?
Which factor(s) is/are mentioned?
Where in the text is this mentioned? (Introduction, specific section on…, etc.)?
How is/are the factor(s) described? (quotation)

#### Analyzing data and distill the findings

Analysis of extracts was conducted based on the Elo and Kyngäs description of deductive content analysis [47], which includes alternating between deductive and inductive phases. The deductively extracted texts containing factors related to migration affecting children’s health were read through by the first author (EW). When reading through the extracts, each text was compared with the framework (see Additional file) and factors included in the text were highlighted. The text was also provided with a code indicating which system level (layer in the framework, see Additional file)  the identified factor was found in. If an extracted text consisted of several factors the text was divided into smaller segments and thereafter coded with a system level. In addition, a note was made regarding within which topic or subheading extracts were identified. This note was used to verify whether the extracts had been coded appropriately. To check for consistency, the last author (MH) independently highlighted factors in a handful of extracts, and a comparison of results showed no major differences between authors (EW, MH). Once all extracts had been worked through, the analysis shifted from a deductive toward a more inductive approach. Extracts coded with the same system level code (i.e., individual level) were read through, and content describing the highlighted factors was condensed. Similar condensed texts were grouped and abstracted into categories describing the content in extracts within each system level. All categories were discussed among the authors until consensus was reached. Shifting back to a deductive approach, all categories were compared with the factors in the framework (see Additional file) to identify overlaps.

## Results

### Characteristics of the included documents

The guidelines consisted of various types of documents, from a one-page document describing how the visual screening was to be conducted to multiple paged instructions on how to conduct the health dialog and what topics to include. References were rarely cited in the documents, although when references were cited, usually the Swedish national guidelines of the SHS were mentioned. An author of the guidelines was rarely named, but the document was often dated and most were dated between years 2017–2021. The health questionnaires consisted mainly of questionnaires addressed to children aged 6, 10, 13 or 14 and 16 years old (i.e., the same ages as when children are invited to health visits). In addition, questionnaires regarding children’s health sent to their parents or caregivers as well as questionnaires addressed to children who were new at the school were included. Whether the questionnaires had been validated in any form was not mentioned in the questionnaires.

### Content in guidelines and health questionnaires

**Fig. 2:**
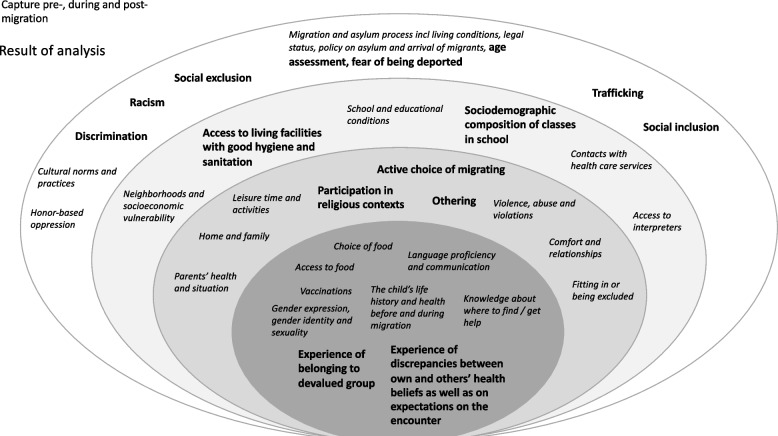
Categories of content of included factors related to migration affecting children’s health in the documents. Non-included factors are marked in bold

In total, 62 percent of the guidelines (*n* = 246) and all 292 health questionnaires included content on factors related to migration affecting children’s health. The categories describing this content of various factors are summarized in Fig. 2 and presented below. In addition, a comparison between the categories (Fig. 2) and the factors in the extraction framework (Additional file 1) shows that most factors are included within the categories of content in the guidelines and health questionnaires. Still, some factors were not found in the content, and these will be presented last in this section.

#### Content at the individual level related to having migrated and health

In the guidelines and health questionnaires the content concerning factors located within the individual level was abstracted into the following categories: *Language proficiency and communication, Knowledge about where to find help, Gender expression, gender identity and sexuality, Food* and *Access to food, Vaccinations,* as well as *The child’s life history and health before and during migration.* The guidelines and questionnaires both included content on *Language proficiency and communication.* In the guidelines this content concerned clarifying the child’s ability to communicate and be understood, language proficiency in Swedish, and ability to read and write. Besides addressing these topics, instructions recommended that adjustments of the health visit should be made by the school nurses based on these clarifications. Similarly, health questionnaires included questions about the languages the child could speak. Content on *Knowledge about where to find help* was also found in both guidelines and questionnaires. In the guidelines, content included instructions about that the school nurse should inform and verify that the child knows where help can be found as well as inform the child about the responsibilities of the school nurse and the school health services. Yet in health questionnaires, questions regarding such knowledge concerned knowing where to find help in case of being violated. The category *Gender expression, gender identity and sexuality* consisted of content in the guidelines highlighting that these topics should be addressed by school nurses asking questions about children’s reflections or thoughts about gender expression, gender identity and sexuality. When included in health questionnaires, such questions included whether the child has anything they would like to share on the topics, as well as if the child is able to be who they are and like who they want. Content concerning *Choice of food* and *Access to food* was found in both guidelines and questionnaires. In the guidelines, food was often mentioned with a single word or couple of words when listing topics to address when talking with children, such as “food”, “eating habits” or “nutrition”. When elaborations were provided, the content concerns what is eaten, when and how often, as well as whether sweets, sweetened beverages or energy drinks are consumed. These aspects were also covered by questions in the health questionnaires. Still, some guidelines also included dietary advice based on the Swedish National Food administration’s recommendations, content on eating habits related to undernutrition and obesity, as well as recommendations concerning fasting during Ramadan. *Vaccination* was also included in the guidelines and health questionnaires. In the guidelines this concerned instructions on clarifying children’s vaccination status and the need for additional vaccinations, whereas health questionnaires included related questions. Regarding *The child’s life history and health before and during migration,* guidelines instructed that the time before and during migration needs to be taken into consideration for children who have recently migrated to Sweden. Overall, children who have recently migrated to Sweden were highlighted in the guidelines as a group requiring additional action and who should be asked specific questions. The guidelines stressed that this group of children should be asked questions about previous or current chronic or infectious diseases, injuries, and symptoms of ill-health. In addition, the guidelines also instructed school nurses to carefully ask about experiences of war, being imprisoned, and occurrences of other trauma. In line with these instructions, the health questionnaires included questions about previous traumatic experience, previous or current chronic or infectious diseases, injuries, and symptoms of ill-health. Still these questions were not only asked to children recently migrated to Sweden but were also included in questionnaires addressed to all children.

#### Content regarding family and social networks related to having migrated and health

The next level collected content on factors related to group level, more specifically family and relationships with others. This content was abstracted into the following categories: *Home and family, Parents’ health and situation, Leisure time and activities, Comfort and relationships contacts, Fitting in or being excluded* and *Violence, abuse, and violations.* Both guidelines and questionnaires addressed *Home and family* with instructions on asking questions aimed at eliciting information about the child’s family, i.e., who is part of the family, who the child lives with and who has custody. In addition, content was also included aimed at finding out more about the home environment and the child’s experiences of feeling comfortable and safe at home. Content in the guidelines regarding *Parents’ health and situation* concerned school nurses being instructed to ask about serious difficulties at home or psychosocial irregularities, as well as the child’s worries about the well-being of family and friends. Furthermore, content in the guidelines highlighted the importance of the parents’ situation regarding socioeconomic status, level of education and social background as well as health in terms of support and conditions, mental ill-health and/or other disease, and passive smoking in the home. Yet the health questionnaires mainly covered the child’s worries about the family’s health and finances as well as whether their parents’ smoke. Content focusing on the children’s feelings of being comfortable in school and their relationships with friends formed the category of *Comfort and relationships*. The importance of children feeling comfortable at school was mentioned in the guidelines, and instructions were provided regarding addressing this in health visits. Asking questions about friends was also stated as important and was sometimes extended to proposing that school nurses ask questions about social situation, social context, and social relations. In the health questionnaires, this was reflected in questions about being comfortable at school, having friends as well as difficulties in relationships with friends. Yet other aspects of social support were also mentioned in the guidelines and questionnaires, such as having an adult or friend to talk to. Having an adult of friend to talk to was included in instructions on asking about if the child feels supported by adults and friends as well as in health questionnaires asking about being supported by parents in important decisions and feeling looked after by adults. As part of understanding the child’s social network, both guidelines and health questionnaires addressed *Leisure time and activities.* School nurses were encouraged by the guidelines to ask about the children’s social activities and activities with friends, although content about leisure time may lack further details and could incorporate anything that is not related to sports. In the health questionnaires such questions included asking about what children do during their leisure time. *Fitting in or being excluded* is a category somewhat related to comfort and social contacts and networks. This category included instructions in the guidelines addressing both whether children feel like they are included in activities and fit in, as well as whether they experience exclusion, loneliness or being picked on, bullied, or hurt by others. The health questionnaires questions focused on asking children about their knowledge of others who are not doing well or being bullied. Content concerning *Violence, abuse and violations* in the guidelines focused on descriptions and questions to address regarding children’s experiences of violence, violence in the home, abuse, honor-based violence, and female genital cutting. In the health questionnaires this was included as questions regarding whether the child has experienced any kind of violations or abuse.

#### Content at the settings level related to having migrated and health

Content on factors related to the settings where children might spend time during their everyday life was gathered in a separate level and abstracted into the following categories: *School and educational conditions, Contacts with the health care services, Neighborhoods and socioeconomic vulnerability,* and *Access to interpreters*. *School and educational conditions* were included in both instructions on what to talk about in the guidelines and health questionnaires. This content and questions concerned children’s experiences of the school environment and of support and help from teachers, whether children felt actively involved in school decisions, and whether they had education adjusted to and relevant to their knowledge level. In the health questionnaires the school environment also concerned questions on being able to focus, completing school assignments on time, and difficulties in the schoolwork. Adding to knowledge about the current situation, the guidelines also highlighted the need to clarify whether children new at the school had previously attended school elsewhere, and their educational level. Similar questions about previous education were also found in health questionnaires given to children new at school. Regarding *Contacts with the health care services,* the health questionnaires contained questions asking about previous and current contacts with any part of the health care services in Sweden or abroad. Instructions on bringing this topic into conversation were also present in the guidelines. The category of *Neighborhoods and socioeconomic vulnerability* was included in the guidelines and contained instructions on asking about where children live, moving between housings, and children’s feelings of safety and security in their neighborhoods. The guidelines also included descriptions of the various living conditions of children in Sweden. The health questionnaires included questions regarding how children live, and moving patterns, although mostly in documents related to migration and to children who are new at the school. The guidelines also provided information highlighting that health is impacted by living in deprived or vulnerable neighborhoods, in families with low socioeconomic status or in poverty. *Access to interpreters* was not related to any particular setting but may influence the interactions at the settings. Questions about needing an interpreter were included in health questionnaires. In the guidelines information on which interpreters to use and in what situations was included, especially when matters of honor-based oppression or female genital cutting were to be discussed.

#### Content at the societal level related to having migrated and health

Content related to factors regarding issues located at a societal level or transcending all levels was gathered and abstracted into categories of *Migration and asylum process, Cultural norms and practices* and *Honor-based oppression.* A category mainly appearing in documents concerning children who have recently migrated to Sweden was *Migration and asylum process*. Both guidelines and health questionnaires focused on clarifying when the child migrated, with whom, along what route, where the child stayed along the way, as well as when the child began the migration. The guidelines highlighted that such questions should be asked, and the health questionnaires contain them. The guidelines also clarified the various legal statuses for persons having migrated that exist in Sweden and what the responsibility of each of the health care services is in relation to this. In addition, questions in the health questionnaires also concerned arrival in Sweden, legal status in Sweden and the reason for migrating. Two categories that are somewhat related and that were sometimes described together in the guidelines are *Cultural norms and practices* and *Honor-based oppression*. Content about *Cultural norms and practices* in the guidelines provided information about the existence of varying cultural norms and practices, norms related to gender, and norms upheld by the Swedish school system, but also how norms and practices relate to honor-based oppression. In the health questionnaires, the cultural norms and practices are recognized in questions about the child’s opportunities for deciding about their life, liking who they want to like, doing what they want to do and spending time with the people they like to spend time with. Furthermore, questions also included whether children feel forced to do things or have to do things they do not want to do. Still, such questions were mainly included in specific health questionnaires given to children regarding *Honor-based oppression* but also in health questionnaires directed at all children. Similarly, *Honor-based oppression* was mainly included in specific guidelines focusing on this topic and/or female genital mutilation, although general guidelines on health visits also included paragraphs on the subject.

#### Factors not included within the content

While the content in guidelines and health questionnaires included most factors related to migration affecting children’s health, some factors were not found in the content (see bold text in Fig. 2). Many of these factors, although illustrated at various system levels, shared a common feature of capturing aspects of *discrimination based on ethnicity or origin*. This category included factors such as the child’s experiences of belonging to a devalued group, experiencing othering, sociodemographic composition of classes in school, discrimination, racism, discrepancies in beliefs and expectations, social exclusion, and social inclusion. In addition, some factors that could be considered as related to the categories of *Migration and asylum process* and *The child’s life history and health before and during migration* were not included. Such factors were choice of migration, experience of trafficking, age assessments, fear of being deported, and access to housing with good hygiene and sanitation.

## Discussion

The results show that municipal and regional guidelines and health questionnaires used in health visits in the Swedish school health services include content on many factors related to migration that affect children’s health (Fig. 2). Thus, the current guidelines and health questionnaires could be considered as relevant for capturing most factors influencing the health of children who have migrated or whose parents have migrated. Yet a question could be asked regarding whether the mere inclusion of these factors is enough to assist school nurses in promoting health and preventing ill-health for these children or if further development of guidelines is needed. In addition, it is important to highlight what the possible implications of not including factors related to migration that affect children’s health might be for these children’s health.

### Current guidelines as support for practice

The results show that content on most factors related to migration that affect children’s health is included as topics to be addressed in health visits in the municipal and regional guidelines and health questionnaires. By addressing these topics, school nurses gather information about the children and their health which guides further health promotion and prevention actions [30]. In addition, the results also show that there are municipal and regional guidelines that provide further information on certain topics, for example concerning food, genital cutting or encounters with children who have recently migrated to Sweden. As school nurses have reported using guidelines and health questionnaire as frameworks for what to address in health visits [29, 30], the inclusion of content on factors related to migration that affect children’s health might ensure that these factors are addressed in clinical practice.

That the guidelines include the factors as topics to be addressed, provides school nurses with room to adjust the conversation on these topics according to their own knowledge and to the specific needs of the individual child. School nurses might thereby use their previous knowledge and experience, which is considered important in guiding and assuring good quality in clinical practice [48, 49]. In addition, using approaches that adjust to the child’s needs, language proficiency and cultural or national background when encountering children who have migrated or whose parents have migrated is described as common among school nurses [50]. The possibility of adjustment is especially important for children who have migrated or whose parents have migrated as experiences, living conditions and health differ greatly within this group of children [1]. Still, such reliance on the individual school nurse to address factors related to migration that affect children’s health in an evidence-based manner without the support of guidelines is problematic [32, 51]. Reliance on individual school nurse’s knowledge might increase dependence on the individual school nurse’s preferences and interests [32]. In addition, uptake of evidence-based practice and research findings if not included in guidelines is sparse among school nurses [51]. Reliance on the individual school nurse might provide large variations in how school nurses conduct clinical practice in health visits, and might therefore increase inequity in care provided.

Hence, the inclusion of the factors as topics to be addressed in health visits could be regarded as a weakness of the documents. The results show that the guidelines often lack further information or specifics on how the school nurses are expected to assess the information gathered by addressing these topics as well as on suggestions of actions to be taken. Such lack of further information guiding clinical practice has also been expressed by Swedish school nurses [15] and in a government official Inquiry on the Swedish SHS [52]. To meet the needs expressed by school nurses [15] and the lack of clinical practice guidelines [52], the inclusion of topics might need to be supplemented by including further information on how to address the factors related to migration, why they are relevant to address in the health visits and if there are certain considerations to be made related to addressing the factors with children who have migrated or whose parents have migrated. The inclusion of further information might be beneficial not only for health visits with children who have migrated or whose parents have migrated, but also for health visits in general. The results show that many of the categories are not uniquely related to migration but rather consist of factors influencing the health of children in general [53, 54]. For example, categories like *Home and family, Parents’ health and situation, Comfort and social contacts,* as well as *Neighborhoods and socioeconomic vulnerability* are similar to what adolescents describe as influencing their health [54]. Thus, the inclusion of further information would strengthen clinical practice in all health visits and support school nurses in conducting health visits addressing factors affecting the health of all children as well as children who have migrated or whose parents have migrated.

### Possible implications of not including all factors that affect children’s health

Although the results show that the categories overlap with many of the factors related to migration affecting children’s health, they also show that some factors are not included in the content in municipal and regional guidelines or health questionnaires. A common denominator for most factors not included in the content is that they could be categorized as related to discrimination based on origin or ethnicity. Hence, these factors relate to discriminatory practices based on the categorizations of people into different groups and the inference of notions about groups being portrayed as more or less valued by society [55, 56]. Such discrimination based on origin or ethnicity is shown to influence children’s health [1, 3, 4, 6, 41–43, 45]. That these factors are not included in guidelines or health questionnaires could imply that they might not be fully addressed in health visits, as school nurses report relying on health questionnaires to address all topics to be included in the health dialog with children [29, 30]. This would imply that children experiencing discrimination based on origin or ethnicity might not be provided with an opportunity of speaking with the school nurse about such experiences and highlighting discriminatory practices affecting their health. This deficiency in municipal and regional guidelines is not compensated for in the national guidelines of the SHS [11], as experiences of discrimination based on origin or ethnicity are not listed as a topic to address in health visits. Still, the national guidelines state that experiences of any sort of discrimination among children should be addressed and preventive efforts against discrimination should be taken, in line with existing regulations regarding non-discrimination [57–59]. In the national guidelines, discrimination is also mentioned in relation to violations as well as equal treatment, which might support an assumption of aspects of discrimination being included in the municipal and regional documents, embedded in the category *Fitting in or being excluded*. For example, arguments could be made regarding school nurses addressing discrimination based on origin or ethnicity when talking about issues of bullying or exclusion during health visits. However, as previously discussed, such reliance on the individual school nurse might be problematic [51]. In addition, the embeddedness of discrimination based on origin or ethnicity might also prevent children from speaking about experiences of discrimination that are not explicitly related to actions by individuals but that rather illustrate systemic or structural discrimination [41].

The results also showed that although content was included on factors related to the categories of *Migration and asylum process* and *The child’s life history and health before and during migration*, there were still similar factors not included in the guidelines and health questionnaires. Yet these factors (choice of migration, experience of trafficking, age assessments, fear of being deported, and access to housing with good hygiene and sanitation) might be included in dialogs during health visits when related content is addressed. Still, if not explicitly stated in the documents, the reliance on the individual school nurse to address these factors increase [32]. Hence, guidelines including all relevant factors related to migration affecting children’s health are needed to ensure the provision of high-quality care that is evidence-based [60] and promotes equity in health.

### Practical implications

The results of this study highlight that content in guidelines and health questionnaires matters and that it is not enough to have guidelines or policy guiding school nursing practice, as most European countries do [14]. Rather, the content of these guidelines needs to be investigated to make sure that they also provide assistance for school nurses in conducting evidence-based practice. In addition, this study also indicates a lack of coherence in guidance for health visits in Sweden, shown by the amount of municipal and regional guidelines and health questionnaires found in this study and confirmed in other studies [32, 52]. Such a lack of coherence indicates geographically-based variations in guidelines that might increase the risk of inequities in care provided. The lack of coherence in municipal and regional guidelines and health questionnaires might be compensated for if the Swedish national guidelines of the SHS would provide extensive guidance of clinical practice. However, the shortage of further information to guide clinical practice found in the regional and municipal guidelines included in this study is not compensated for by content in the Swedish national guidelines of the SHS. The national guidelines only provide school nurses with further descriptions related to a few of the categories found in the regional and municipal content, such as eating habits and vaccinations [11]. Furthermore, the national guidelines are criticized for not providing sufficient evidence-based guidance on health promoting and preventive actions [52]. Hence, shortages in content in guidelines and health questionnaires at municipal and regional levels might reflect the content at national level, indicating that revision and development at all three levels is warranted. Inspiration for development of clinical practice guidelines for school nursing could be provided through efforts made by the school nursing association in the US [22, 60]. Yet recommendations have already been made to develop the Swedish national guidelines of the SHS [52]. Based on the results of this study, such revision and development of the Swedish national guidelines of the SHS would benefit from including all factors related to migration affecting children’s health. A revision and development could also introduce further information assisting school nurses in conducting evidence-based practice when addressing these factors. Including such content in the national guidelines would also ensure that school nursing all over Sweden is guided by the same knowledge foundation, thereby promoting equity in provision of health care. In addition, by using the results of this study, school nurses might increase their awareness about possible improvements needed in their guidelines as well as the importance of including all factors related to migration affecting children’s health in guidelines of health visits.

### Limitations

To enhance quality and rigor, the READ approach of Dalglish et al. [35] was used to conduct the document analysis. Yet limitations of the study should be acknowledged. Response rates show that not all municipalities chose to respond and submit materials, implying that the results do not provide a complete overview of all content in municipal and regional guidelines and health questionnaires used in health visits in Sweden. However, all municipalities were invited and at least two municipalities in each region in Sweden responded. In addition, the respondents contained a mix of urban and rural municipalities from all over the country. Such variation could indicate that the study findings might be generalizable for and transferrable to SHS in all municipalities in Sweden. To further enhance the research quality [61], validation of coding by the last author (MH) and discussion among authors was conducted to ensure the data was not misrepresented, and to reflect critically on interpretations made. Still, the interpretation of data and reporting of findings are the work of the authors and may hence be biased toward their perspectives, regardless of efforts taken to ensure quality.

## Conclusions

Municipal and regional guidelines and health questionnaires used in health visits in the Swedish school health services include content on factors related to migration affecting the health of children. Still, the guidelines do not provide detailed guidance on how these factors should be addressed, nor do the guidelines and health questionnaires address all relevant factors. The non-inclusion of factors related to discrimination based on ethnicity or origin, together with a lack of detailed guidance highlights that further development of guidelines at national, regional and municipal levels is warranted.

## Supplementary Information

**Additional file 1.** Identified factors related to migration affecting children’s health.

## Data Availability

The dataset generated by this study is available in the figshare repository, 10.6084/m9.figshare.23500767.v1.

## Abbreviation

SHS: School health services
