# Supplementary material for: In search of factors related to migration affecting children’s health – an analysis of documents guiding health visits within the Swedish school health services
Source: Arch Public Health. 2023 Jun 13;81:103. doi: 10.1186/s13690-023-01125-z (PMC10262379; doi:10.1186/s13690-023-01125-z)
Supplement: Supplementary file 1 — Additional file 1. Identified factors related to migration affecting children’s health. [file 13690_2023_1125_MOESM1_ESM.docx]

Additional file 1

**Identified factors related to migration affecting children’s health**

| ***Reference*** | ***Pre migration*** | ***During migration*** | ***Post migration*** |
| --- | --- | --- | --- |
| Abubakar et al. (2018) | Violence  Discrimination  Poverty  Choice of migration | Violence (especially for unaccompanied children)  Sexual violence  Discrimination  Increase risks of violence, assault and abuse for LGBTQ+  Trafficking  The asylum process  Poverty  Access to health care services  Access to food and food quality | Racism  Discrimination  The asylum process  Poverty  Access to food and food quality  Disrupted education  Support from teachers  Adaption of materials and to language proficiency in school  Lower quality of education  Bullying  Social exclusion  Asylum process related insecurities  Getting an education  Positive experience of school and education  Family situation  Having friends  Cultural practices such as forced marriage, genital cutting, and historical and cultural norms, including unfamiliarity with or restricted use of gynecological and obstetric care  Gender-based violence and honor crimes |
| Aldrige et al. (2020) |  |  | Infectious diseases  Violence  Assaults  ”mortality advantage” |
| Carrasco-Sanz et al. (2017) |  | Access to health care services  Trafficking | Lower vaccination coverage  Language barriers in health care services  Health needs: vaccination, nutrition advice, injury prevention advice, socioemotional development/ attachment, vision, hearing, oral health, trauma, screen for poverty related illnesses. |
| Chiarenza et al. (2019) |  |  | Legal status in new country  Language barriers  Knowledge on available health care services |
| Curtis et al. (2018) |  | Trauma and stressful events | Adherence to nutritional guidelines  Consumption of fruit and vegetables  Consumption of sweetened beverages and sweets  Citizenship/legal status  Moving between places and schools  Insecurities in social, material and financial status  Loss of social networks  Loneliness  Discrimination  Experience of cultural dissonance and belonging to a devalued group  Dissonance between beliefs in health between migrants and health professionals  Participation in social activities  Comfort at home |
| Dawson-Hahn et al. (2020) |  |  | Access to organic produce  Safety of being outdoors  Eating habits |
| Folkhälsomyndigheten (2019)  reference no 4 |  |  | Being badly treated because of my background  Having enough money for expenses  Family finances  Language proficiency  Keeping up with schoolwork  School-related stress  Being afraid of peers  Bullying  Safety in neighborhood  Positive experience of school and education  Comfort in school  Social inclusion  Fitting in among peers  Knowledge on where to find help |
| Folkhälsomyndigheten (2019)  reference no 5 | Experience of war | Migrating alone (unaccompanied) | Needs of vaccinations |
| Granvik-Salminathen (2020) |  |  | Sociodemographic composition of classes in school  “fitting in” among peers |
| Greensmith et al. (2018) |  |  | Experience of structural racism  Experience of “fitting in”  Dissonance in expectations on encounters between migrants and health professionals  Proficiency in majority language  Placed in lower class at school due to low proficiency in majority language  Coping with othering  Being able to share life story without adapting to others |
| Harding et al. (2015) |  |  | Structural adversity such as racism and lower socioeconomic status  Parents care and support  Participation in religious gatherings |
| Hilario et al. (2015) | country of origin and level of economic development in  country of origin, |  | immigrant generation, length of stay and age at migration,  place of resettlement and urban residence,  parental ability to speak one official language,  family income, family relationships, school and neighborhood  environment, experiences of harassment and racism, experiences of uprooting and disconnection, and sex. |
| Hjern (2012) | Prevalence of infectious diseases | Experiences of delayed asylum processes | Parents having low income  Single parents  Living in low-status neighborhood  Exposure of passive smoking |
| Kadir et al. (2019) | Female gender mutilation | Children separated or travelling unaccompanied  (UASC) are particularly vulnerable  Access to housing with good hygiene and sanitation  Traumatic events such as torture, sexual violence or kidnapping  Injuries that are not treated  Infection with  drug-resistant organisms.  Malnourishment  Transit and host country reception policies also impact  the mental health outcomes of children on the move. | Structural, financial, language and cultural barriers in access to healthcare affect  Access to housing with good hygiene and sanitation  Vaccination needs  Dissonance in health beliefs  Access to health care services  Maternal PTSD and depression are correlated with increased risk of PTSD, PTS symptoms,  behavioral problems and somatic complaints in their children.  Racism and xenophobia play an important role in the psychological health and well-being of children on the move.  Good caregiver mental health is a protective factor for the mental and behavioral health  of refugee children.  Social inclusion  Supportive families and environments  Parents mental health  Positive experiences of school  Female gender mutilation |
| Khawaja et al. (2018) |  |  | Social support  Feeling accepted and respected  Feeling included  Support in school |
| Lebano et al. (2020) |  |  | Sociodemographic conditions |
| WHO (2018) | • Chronic infectious  agents  • Violence (including  armed conflict and  political persecution)  • Lack of health and  dental care  • Food insecurity  War, seeking refuge from violence or crimes against human rights | Exposure at sea  • Injuries  • Hunger  • Acute infectious  disorders  • Food insecurity  • Incarceration  • Separation from  caregivers  • Trafficking  • Exploitation  • Violence  • Lack of health and  dental care  Routes of migration presents varying risks  Being hold in captivity  Separation from parent  Inadequate housing, poor hygiene, and sanitation | Barriers to accessing  care  • Barriers to accessing  education  • Social marginalization  and isolation  • Inadequate and  unstable housing  • Daily stressors  •Discrimination/bullying  • Threat of deportation  • Children left behind  • Caregivers' mental  health problems  • Exploitation  Uncertainty due to prolonged asylum processes  Moving repeatedly  Inadequate housing,  Poor living conditions  Social isolation  Access to school and social activities  Parents stress and pressure to make ends meet (financially)  Racism  Language proficiency  Cultural differences and new environment provides difficulties in encounters with health care services  Fear of being deported  Risk of D-vitamin deficiency  Socioeconomic deprivation  Parental divorce Bullying,  Parental mental health  Migrating alone  Age assessments  Newly settled migrants face numerous barriers for accessing care: unfamiliarity with rights, entitlements and the overall health system; gaps in health literacy; social  exclusion; and direct and indirect discrimination.  Cultural and language  barriers can also influence the quality of the care received.  Access to interpreters  Parental support  Access to schools  Development of social networks  Facilitate contacts with families/children with the  same origin  Provide basic material resources  Prevent xenophobia  Provide culturally sensitive parent-support  programs  Avoid relocation  Ensure parental access to psychiatric care  Have family reunification policies that minimize  children becoming left behind  Avoid detention of children  Provide child-friendly spaces  Treat trauma with sensitive care and education  Ensure early entry into pre-school and school  Provide continuity of care for unaccompanied minors  Use a holistic age assessment policy |

**Framework of factors related to migration affecting children’s health.**

The framework was used in extraction of text describing factors related to migration affecting children’s health identified in previous research (Abubakar et al., 2018; Aldrige et al., 2020; Carrasco-Sanz et al., 2017; Chiarenza et al., 2019; Curtis et al., 2018; Dawson-Hahn et al., 2020; Folkhälsomyndigheten, 2019a; Folkhälsomyndigheten, 2019b; Granvik-Salminathen, 2020; Greensmith et al., 2018; Harding et al., 2015; Hilario et al., 2015; Hjern, 2012; Kadir et al., 2019; Khawaja et al., 2018; Lebano et al., 2020; WHO, 2018)

.
